# User engagement in relation to effectiveness of a digital lifestyle intervention (the HealthyMoms app) in pregnancy

**DOI:** 10.1038/s41598-022-17554-9

**Published:** 2022-08-13

**Authors:** Pontus Henriksson, Jairo H. Migueles, Emmie Söderström, Johanna Sandborg, Ralph Maddison, Marie Löf

**Affiliations:** 1 Department of Health, Medicine and Caring Sciences, Linköping University, 581 83 Linköping, Sweden; 2 Department of Biosciences and Nutrition, Karolinska Institutet, Huddinge, Stockholm, Sweden; 3 Institute for Physical Activity and Nutrition, Deakin University, Geelong, Australia

**Keywords:** Disease prevention, Nutrition, Public health, Medical research

## Abstract

Although user engagement is generally considered important for the effectiveness of digital behavior change interventions, there is a lack of such data in pregnancy. The aim of this study was therefore to examine the associations of user engagement with the HealthyMoms app with gestational weight gain, diet quality and physical activity in pregnancy. The study involved secondary analyses of participant data from the intervention group (n = 134) in a randomized controlled trial to determine the effectiveness of a 6-month mHealth intervention (the HealthyMoms app) on gestational weight gain, diet quality and physical activity. In adjusted regression models, the total number of registrations from three self-monitoring features (i.e., for weight-, diet- and physical activity) was associated with lower gestational weight gain (β =  − 0.18, *P* = 0.043) and improved diet quality (β = 0.17, *P* = 0.019). These findings were mainly attributable to the associations of physical activity registrations with lower gestational weight gain (β =  − 0.20, *P* = 0.026) and improved diet quality (β = 0.20, *P* = 0.006). However, the number of app sessions and page views were not associated with any of the outcomes. Our results may motivate efforts to increase user engagement in digital lifestyle interventions in pregnancy. However, additional studies are needed to further elucidate the influence of different types of user engagement in digital pregnancy interventions on their effectiveness.

Trial registration: ClinicalTrials.gov (NCT03298555); https://clinicaltrials.gov/ct2/show/NCT03298555 (date of registration: October 2, 2017; date of first enrolled participant: October 24, 2017).

## Introduction

Excessive gestational weight gain is common worldwide and affects about 50% of women in the USA and Europe^1,2^. Preventing excessive gestational weight gain is of importance as it is a risk factor for several adverse pregnancy outcomes such as preeclampsia, gestational diabetes, cesarean delivery, and macrosomia^3,4^. Systematic reviews and meta-analyses have shown that interventions (generally traditional face-to-face interventions and supervised exercise sessions), intended to promote healthy diet and physical activity can prevent excessive gestational weight gain^5,6^. For instance, a Cochrane review and meta-analysis of 24 studies and 7096 participants showed that diet and/or exercise interventions reduced the risk of excessive gestational weight gain by 20% on average (risk ratio 0.80, 95% confidence interval 0.73 to 0.87)^5^.

Recently, interest in mobile health (mHealth) interventions to promote healthy weight gain, diet and physical activity in pregnancy has increased^7^. mHealth interventions have several advantages compared to more traditional in-person delivered intervention programs in terms of reach and potential cost-effectiveness^8^. Although engagement with and effectiveness of exclusively digital mHealth interventions vary considerably between studies, some interventions have shown promise to reduce gestational weight gain and promote a healthy diet during pregnancy^7–10^. For instance, our previous randomized controlled trial, the HealthyMoms trial, showed that access to a smartphone app in pregnancy reduced weight gain in women with overweight or obesity before pregnancy and improved diet quality irrespectively of pre-pregnancy body mass index (BMI)^9^.

Engagement in digital behavior change interventions has been defined as the “extent (i.e., amount, frequency, duration, and depth) of usage” but also as a “subjective experience characterized by attention, interest and affect”^11^. Although user engagement in smartphone interventions generally has been associated with intervention effectiveness in non-pregnant adults^12^, there is a great lack of such studies in pregnancy. To the best of our knowledge, no previous mHealth study has examined how engagement with an exclusively digital intervention is associated with effectiveness on gestational weight gain and health behaviors (e.g., diet, physical activity) in pregnancy. Such information is essential for understanding the role of the amount and type of user engagement with intervention effectiveness in digital interventions. Ultimately, promotion of more beneficial user engagement patterns (i.e., optimal amount and type of engagement) may result in more effective digital lifestyle interventions in pregnancy. Hence, further studies are needed to elucidate the role of engagement for the effectiveness of digital lifestyle interventions in pregnancy. We therefore utilized data from the HealthyMoms trial^9^ with the aim to examine associations of app user engagement with gestational weight gain, diet quality and physical activity.

## Methods

### Study design and participants

This study involved secondary analyses of participant data from the intervention group (n = 134) in a randomized controlled trial to determine the effectiveness of a 6-month mHealth intervention (the HealthyMoms app) on gestational weight gain, diet quality and physical activity. Detailed information regarding the HealthyMoms trial and results for the effectiveness of the intervention have been published elsewhere^9,13^. Briefly, 305 women were randomized to either the intervention (n = 152) or control group (n = 153) after performing the baseline measurements in gestational week 14 (13.8 ± 0.6 gestational weeks). Women randomized to the control group received standard antenatal care, while women in the intervention group received standard antenatal care as well as the HealthyMoms app. At follow-up in gestational week 37 (36.4 ± 0.4 gestational weeks), a total of 271 women (89% of the original sample) were measured. To address the aim of the present study, user engagement data from women randomized to the HealthyMoms app (n = 134) were used.

### The HealthyMoms app and user engagement data

**Figure 1:**
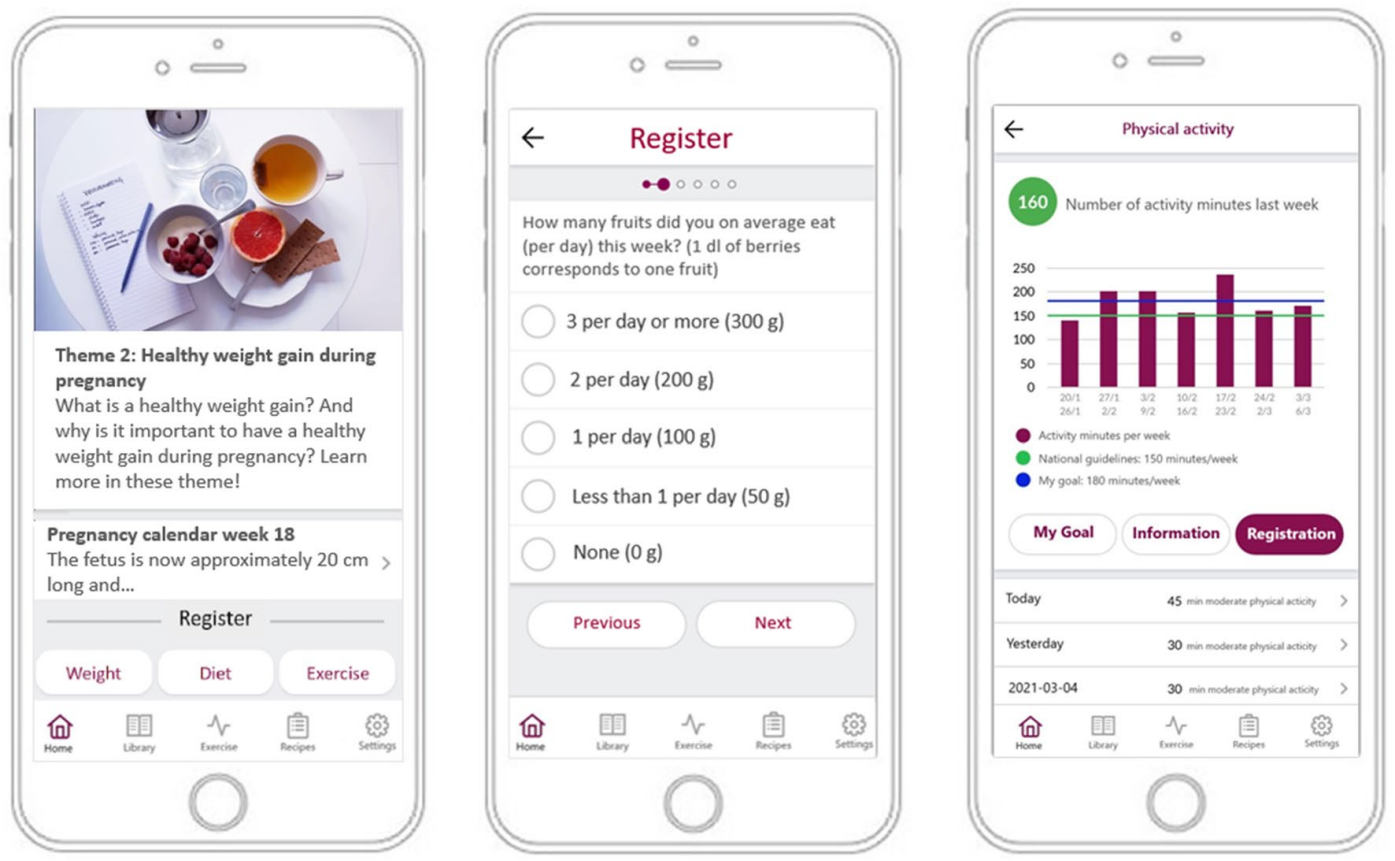
Screenshots of the HealthyMoms app (translated from Swedish to English). Left panel: Homepage of the HealthyMoms app with access to the registrations of the self-monitoring features for weight-, diet- and physical activity; Middle panel: Example of the diet registration feature; Right panel: Example of feedback for physical activity registrations.

The HealthyMoms app is a comprehensive program aimed to promote healthy weight gain, diet and physical activity in pregnancy^9,13^. Briefly, the app has seven features, which include: (1) informational themes that change every second week; (2) three self-monitoring features in which participants could register their weight, diet, and physical activity throughout the intervention period; (3) push notifications; (4) an exercise guide (e.g., aerobic and resistance exercises, training programs and videos); (5) recipes; (6) a pregnancy calendar; and (7) an app library (e.g., frequently asked questions, practical tips). Women were asked to register their weight, diet and physical activity according to their own preference. Figure 1 present screenshots from the HealthyMoms app to visualize the homepage and the registration features. Women were asked to wear an accelerometer (for the physical activity measurement) 1–2 weeks before the follow-up measurement in gestational week 37. Therefore, we selected data for the first 20 weeks of app usage (i.e. the first 10 themes).

We considered two types of engagement data in the current study (registrations and app usage). First, we utilized data from the HealthyMoms app to obtain the number of registrations from the self-monitoring features for weight-, diet- and physical activity registrations (hereafter referred to as registrations) (n = 134) for the first 20 weeks of app usage. We considered the number of weight-, diet- and physical activity registrations separately but also the total number of registrations from all three categories. Second, through Google Analytics, we also utilized data regarding the app usage assessed as the number of app sessions (which represent a single period of user interaction with the app) and page views from the first 20 weeks of the intervention. We were able to utilize Google Analytics data for the women that started the intervention in January 2019 and onwards (n = 60). Five women did not have any registrations and thus the Google Analytics sample consisted of 55 women. We only included data regarding app sessions and page views from sessions that were ≥ 5 s to exclude unrealistically short and likely unintentional sessions.

### Effectiveness outcomes

Outcome measures were evaluated at both the baseline and follow-up measurements and have been described in detail previously^9,13^. In brief, body weight was measured in underwear using standardized procedures. Consequently, gestational weight gain was calculated as the body weight at the follow-up measurement minus the body weight at baseline. Women were classified as having excessive gestational weight gain using the cut-offs from the National Academy of Medicine, USA (formerly Institute of Medicine) for the recommended weight gain in the second and third trimester according to pre-pregnancy BMI groups (underweight: ≥ 0.59 kg per week; normal weight: ≥ 0.51 kg per week; overweight: ≥ 0.34 kg per week; and obesity: ≥ 0.28 kg/week)^14^. Diet was measured using Riksmaten FLEX which has been developed by the Swedish National Food Agency^15^. This method utilizes web-based data collected from three repeated 24-h dietary recalls which was then linked to the Swedish National Food Composition Database. Diet quality was assessed using a Swedish Healthy Eating Index Score which is based on the Nordic Nutrition Recommendations^9,16^. The score consists of 9 components; (1) fruit and vegetables; (2) fish and shellfish; (3) red meat; (4) fiber; (5) wholegrain; (6) polyunsaturated fat; (7) monounsaturated fat; (8) saturated fat; and (9) sucrose. Each item could yield a score between 0 and 1 and thus the total score could range between 0 and 9 with higher values indicating better diet quality. Physical activity was assessed using an ActiGraph wGT3x-BT accelerometer (ActiGraph, Pensacola, FL). The women were instructed to wear the accelerometer at the non-dominant wrist 24 h a day for 7 consecutive days. Women that could not wear the accelerometer at the wrist (e.g., due to hygiene restrictions at work) were instructed to wear the accelerometer at the hip (baseline, n = 9; follow-up, n = 8). Data was collected at 100 Hz and participants filled in a diary to record non-wear and sleep time. Women with at least one valid day were included in the analysis and a valid day was defined as ≥ 1/3 of the of the 24-h period being wear time, ≥ 2/3 of the wake time being wear time, and ≥ 2/3 of the sleep time being wear time^9^. The cut-offs by Hildebrand et al. (i.e., wrist: 100 mg; hip: 70 mg) were applied to define moderate-to-vigorous physical activity^17^. Daily moderate-to-vigorous physical activity was calculated as the weighed mean of weekdays and weekends days^9^. Physical activity data processing was performed using the R software program (v. 4.1.0, https://www.cran.r-project.org/)^18^ and the GGIR package^19^.

### Covariates

At the baseline measurement, participants completed a questionnaire regarding their age, educational attainment, and the number of children they had given birth to (parity). Furthermore, women responded to questions regarding their perceived competence to have a healthy diet and physical activity. Questions were based on the original questionnaires of perceived competence^20,21^ and were modified to assess diet and physical activity (a translation of the questionnaire is presented in Table S1).

### Statistical analysis

Differences in user engagement by user characteristics (i.e. age, educational attainment, parity, pre-pregnancy BMI and perceived competence) were examined by the Mann–Whitney U test since these variables were not normally distributed (all *P* < 0.05 using the Kolmogorov–Smirnov test). Associations of user engagement with the outcome variables gestational weight gain, diet quality and physical activity were examined by linear regression and two regression models were created. Model 1 included only an adjustment for the baseline value for the outcome variable (analyses with gestational weight gain were adjusted for baseline BMI). Model 2 were adjusted for age, parity (0 vs. ≥ 1), educational attainment (high school vs. university degree), perceived competence for healthy diet and physical activity at baseline, baseline BMI as well as the baseline value for the outcome variable (for analyses with the Swedish Healthy Eating Index score and moderate-to-vigorous physical activity). The HealthyMoms trial was dimensioned to detect a 1.5 kg difference in gestational weight gain between the intervention and control group with 80% power (α = 0.05, two-tailed). In this secondary analysis, sample sizes of 134 and 55 women provide 80% power (α = 0.05, two-tailed) to observe standardized regression coefficients of 0.24 and 0.36, respectively. No violations regarding the assumptions of the regression models were observed^22^. A two-tailed *P* value of < 0.05 was considered statistically significant and the statistical analyses were conducted in SPSS (IMB SPSS statistics, version 26, IBM Corp., NY, USA) and R (v. 4.1.0, https://www.cran.r-project.org/).

### Ethics approval and consent to participate

The HealthyMoms trial received approval from the Regional Ethical Review Board in Linköping, Sweden (reference numbers 2017/112-31 and 2018/262-32). All women provided written informed consent before entering the trial.

## Results

### Participants’ characteristics and user engagement data

**Figure 2:**
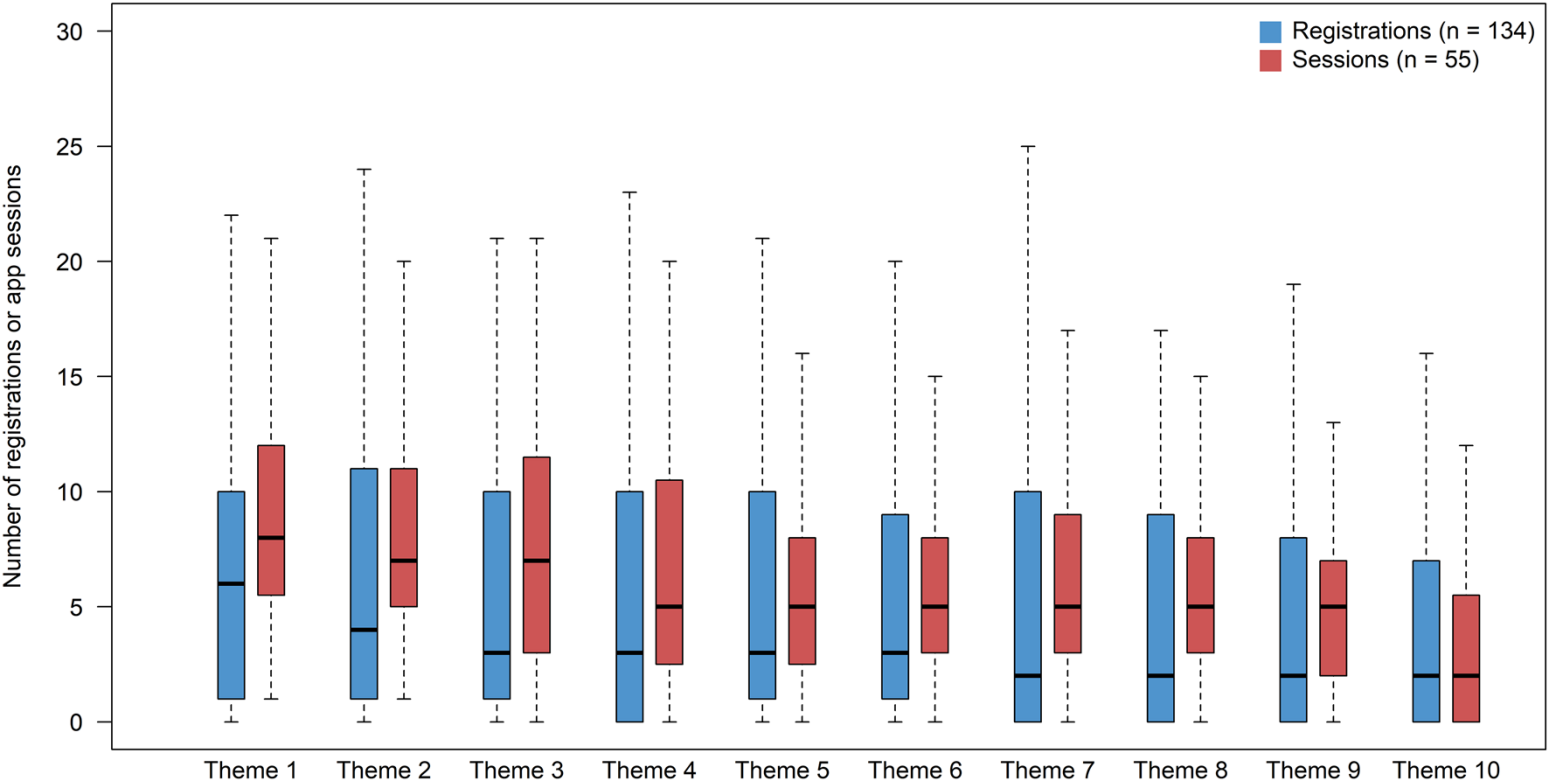
Number of registrations and app sessions per theme using box-and-whiskers plots. Registrations refers to the total number of registrations from the self-monitoring features for weight-, diet- and physical activity within the HealthyMoms app whereas an app session represents a single period of user interaction with the app. The box visualizes values for the first quartile, median and third quartile whereas the whiskers represent the first quartile minus 1.5 times the interquartile range and the third quartile plus 1.5 times the interquartile range. Each theme represents a two-week period.

Participants’ characteristics and overall user engagement data are presented in Table 1. Additional descriptive data of participants in the intervention and control group is presented in Table S2. Overall, user engagement was high with the average number of app sessions and registrations being 66 and 63, respectively over the 20 weeks. Figure 2 shows user engagement over time, presented as the number of app sessions and registrations for each of the 10 two-week themes (detailed data in Table S3). Although engagement decreased over time, overall engagement remained relatively high throughout the intervention period. For instance, at least 60% of participants had one or more app session or registration for each theme (two-week period) throughout the intervention.

**Table 1:** Descriptive statistics for participants’ characteristics and user engagement with the HealthyMoms app (n = 134).

	n	Value^a^	Min–Max
**Gestational week 14**			
Age (y)	134	31.5 (4.2)	22–44
Educational attainment	134		
High school (12 y), (% [n])		22.4% (30)	
University degree, (% [n])		77.6% (104)	
Parity	134		
0, (% [n])		58.2% (78)	
≥ 1, (% [n])		41.8% (56)	
Perceived competence for healthy diet (points)	134	21.9 (4.7)	8–28
Perceived competence for healthy PA (points)	134	20.3 (5.6)	5–28
Height (cm)	134	166 (6)	146–182
Weight (kg)	134	68.1 (12.9)	44.7–120.0
BMI (kg/m^2^)	134	24.6 (4.3)	17.4–41.1
Swedish Healthy Eating Index score (points)^b^	133	6.51 (0.99)	3.71–8.74
Moderate-to-vigorous PA (min/day)^c^	130	38.8 (24.2)	3.2–121.6
**Gestational week 37**			
Weight (kg)	134	78.7 (13.1)	54.8–129.6
BMI (kg/m^2^)	134	28.5 (4.2)	21.3–43.6
Swedish Healthy Eating Index score (points)	133	6.52 (0.93)	3.62–8.38
Moderate-to-vigorous PA (min/day)	131	26.3 (19.0)	0.5–90.8
**Change between gestational week 14 and 37**			
Gestational weight gain (kg)	134	10.6 (3.3)	0.8–20.9
BMI (kg/m^2^)	134	3.8 (1.2)	0.3–7.2
Swedish Healthy Eating Index score (points)^b^	132	0.01 (1.10)	− 2.40–3.64
Moderate-to-vigorous PA (min/day)^c^	127	− 12.6 (20.9)	− 71.0–34.8
**Engagement within the HealthyMoms app**^**d**^			
All registrations^e^ (no.)	134	62.7 (60.8)	0–270
Weight registrations^e^ (no.)	134	17.8 (18.3)	0–135
Diet registrations^e^ (no.)	134	5.9 (7.6)	0–39
Physical activity registrations^e^ (no.)	134	39.0 (49.8)	0–231
App sessions^f^ (no.)	55	66.3 (40.7)	7–184
Page views (no.)	55	847 (494)	85–2160

*BMI* Body mass index, *PA* Physical activity, *SD* Standard deviation.

^a^Values are mean (SD) for continuous variables or % (n) for categorical variables.

^b^The Swedish Healthy Eating Index is based on 9 items (1) fruit and vegetables; (2) fish and shellfish; (3) red meat; (4) fiber; (5) wholegrain; (6) polyunsaturated fat; (7) monounsaturated fat; (8) saturated fat; (9) sucrose producing a score ranging from 0 to 9.

^c^Measured using accelerometry as described in the method section.

^d^User engagement during the first 20 weeks of the intervention.

^e^Refers to the number of registrations from the self-monitoring features for weight-, diet- and physical activity within the HealthyMoms app.

^f^An app session represents a single period of user interaction with the app.

### Correlates of user engagement

User engagement according to participant characteristics is presented in Table 2. There were no statistically significant differences in the number of app sessions and registrations according to age, educational attainment, and perceived competence for a healthy diet. However, women with a perceived competence of healthy physical activity above the median had more registrations than corresponding women below the median (*P* = 0.003). Furthermore, women that were primiparous (i.e., expecting her first child) or had a BMI < 25.0 kg/m^2^ had a greater number of app sessions than women that already had children or had a BMI ≥ 25.0 kg/m^2^ before pregnancy, (*P* = 0.032 and 0.041, respectively).

**Table 2:** Differences in app user engagement according to characteristics of the women.

Characteristics	Number of registrations^a^			Number of app sessions^b^		
	n	Median (IQR)	*P*^c^	n	Median (IQR)	*P*^c^
**Age**						
18–29 y	50	30 (94)		22	68 (66)	
≥ 30 y	84	45 (95)	0.32	33	54 (41)	0.19
**Educational attainment**						
High school	30	27 (102)		11	51 (45)	
University	104	39 (96)	0.96	44	60 (44)	0.45
**Parity**						
0	78	44 (97)		35	62 (59)	
≥ 1	56	27 (82)	0.17	20	39 (48)	0.032
**Pre-pregnancy BMI**						
< 25.0 kg/m^2^	93	44 (89)		39	68 (57)	
≥ 25.0 kg/m^2^	41	24 (110)	0.31	16	41 (29)	0.041
**Perceived competence for healthy diet**						
Below median	63	33 (93)		27	60 (66)	
Above median	71	38 (103)	0.85	28	59 (32)	0.91
**Perceived competence for healthy PA**						
Below median	64	25 (65)		23	48 (51)	
Above median	70	66 (103)	0.003	32	68 (55)	0.079

*IQR* Interquartile range, *BMI* Body mass index, *PA* Physical activity.

^a^Refers to the number of registrations from the self-monitoring features for weight-, diet- and physical activity within the HealthyMoms app.

^b^An app session represents a single period of user interaction with the app.

^c^Refers to the *P* value of a Mann–Whitney U test.

### Associations of user engagement with gestational weight gain, diet and physical activity

Table 3 presents associations between user engagement and gestational weight gain as well as change in diet quality and physical activity between gestational week 14 and 37. The total number of registrations (the sum of weight, diet and physical activity registrations) was associated with improved diet quality (β = 0.16, *P* = 0.025) in model 1 and with both lower gestational weight gain (β =  − 0.18, *P* = 0.043) and improved diet quality (β = 0.17, *P* = 0.019) in model 2. The number of physical activity registrations was associated with lower gestational weight gain (β =  − 0.20, *P* = 0.026) and improved diet quality (β = 0.20, *P* = 0.006) in model 2, whereas no such statistically significant associations were observed for weight and diet registrations. However, there were no statically significant associations of the number of app sessions or page views with gestational weight gain or change in diet quality and moderate-to-vigorous physical activity in any of the regression models.

**Table 3:** Associations of app user engagement with gestational weight gain and change in diet quality and physical activity between gestational week 14 and 37.

Engagement data	Gestational weight gain (kg)		Change in Swedish Healthy Eating Index (points)		Change in moderate-to-vigorous PA (min/day)	
	β	*P*	β	*P*	β	*P*
**Total registrations**^**a**^						
Model 1^b^	− 0.13	0.13	0.16	0.025	0.04	0.59
Model 2^c^	− 0.18	0.043	0.17	0.019	0.00	0.98
**Weight registrations**^**a**^						
Model 1^b^	− 0.01	0.92	− 0.01	0.89	0.02	0.80
Model 2^c^	− 0.02	0.85	− 0.03	0.72	− 0.02	0.77
**Diet registrations**^**a**^						
Model 1^b^	− 0.06	0.50	0.09	0.19	0.00	0.98
Model 2^c^	− 0.09	0.33	0.12	0.087	− 0.04	0.61
**Physical activity registrations**^**a**^						
Model 1^b^	− 0.15	0.087	0.18	0.009	0.04	0.57
Model 2^c^	− 0.20	0.026	0.20	0.006	0.02	0.83
**Number of app sessions**^**d**^						
Model 1^b^	− 0.04	0.80	0.06	0.59	− 0.23	0.051
Model 2^c^	− 0.11	0.53	0.08	0.54	− 0.25	0.069
**Number of page views**						
Model 1^b^	− 0.18	0.21	0.07	0.53	− 0.19	0.096
Model 2^c^	− 0.25	0.13	0.10	0.43	− 0.23	0.094

*β* Standardized regression coefficient, *BMI* Body mass index, *PA* Physical activity.

^a^Refers to the number of registrations from the self-monitoring features for weight-, diet- and physical activity within the HealthyMoms app.

^b^Adjusted for baseline value for the outcome variables (analyses with gestational weight gain was adjusted for baseline BMI).

^c^Adjusted for age, parity, educational attainment, baseline perceived competence for healthy diet and physical activity, baseline BMI as well as baseline value for the outcome variable (for analyses with Swedish Healthy Eating Index and moderate-to-vigorous PA).

^d^An app session represents a single period of user interaction with the app.

We conducted supplementary and sensitivity analyses to further assess the robustness of our results. First, we conducted a logistic regression to examine the associations of user engagement with the odds of excessive gestational weight gain (Table S4). In general, results were quite comparable to our main results (i.e. when analyzing gestational weight gain as a continuous variable). For instance, in model 2, the odds ratios for excessive gestational weight gain per 1 SD increase in total registrations and physical activity registrations were 0.67 (95% CI: 0.44–1.02) and 0.61 (95% CI: 0.39–0.95), respectively. Second, we explored whether associations for physical activity were comparable if only including participants with wrist data and ≥ 4 valid days (including ≥ 1 weekend day) of accelerometer data, however estimates remained comparable to our main results.

## Discussion

### Main results

The aim of the current study was to examine the associations of user engagement with the HealthyMoms app with gestational weight gain, diet quality and physical activity in pregnancy. The main finding was that a greater number of registrations within the HealthyMoms app was associated with lower gestational weight gain and improved diet quality. These results were mainly attributable to the number of physical activity registrations, which were associated with gestational weight gain and diet quality. However, the number of app sessions and page views were not associated with gestational weight, diet quality and physical activity.

### User engagement

Previous stand-alone mHealth interventions in pregnancy have in general reported relatively high levels of user engagement as indicated by app usage, registrations of weight and physical activity and response rates to messages^8,10,23,24^. Conversely, online interventions (generally consisting of access to a webpage)^25,26^ and multi-component interventions^27,28^ (where mHealth interventions have been provided in conjunction to face-to-face interventions) have generally showed lower engagement. Thus, it appears that user engagement is higher in stand-alone mobile phone interventions than online and multicomponent ones, which may be due to several reasons. It may be speculated that app engagement may be lower in multi-component interventions if participants are already satisfied with the intervention content delivered in the non-mHealth parts of the intervention. Furthermore, an app may increase user engagement compared to a website since an app may be more easily accessible and often include push notifications which remind users to engage with the app.

Few studies have examined whether user engagement in digital interventions in pregnancy differs according to user characteristics. A previous study in the US reported that women of ethnic minorities and with low income generally had lower levels of engagement in an online intervention^29^. Our results observed no differences in user engagement according to educational attainment, age, and perceived competence for a healthy diet. However, there was some evidence that women that were multiparous, overweight before pregnancy or had a low perceived competence for healthy physical activity had lower engagement with the HealthyMoms app. These findings may be compared with data from non-pregnant populations which have reported positive associations with self-efficacy and user engagement and inverse associations between body weight and user engagement^11^. Furthermore, our qualitative evaluation of the HealthyMoms app^30^ showed that multiparous women in general had a lower need of pregnancy-related content in the app and less time to spend on such an app. Nevertheless, further studies are needed to identify and understand differences in user engagement and how they influence effectiveness in digital interventions during pregnancy.

### Associations of user engagement with intervention effectiveness

This is, to the best of our knowledge, the first study to examine associations of user engagement with the effectiveness of a stand-alone smartphone app on weight gain, diet quality and physical activity in pregnancy. However, our results may be compared with the PEARS randomized controlled trial which examined the effectiveness of a multi-component intervention that included an antenatal behavior change intervention combined with an app^27^. When the authors analyzed differences of a comprehensive set of dietary and physical activity variables between users and non-users of the app, only the glycemic index and the proportion of energy from free dietary sugars were statistically significantly lower among app users (*P* = 0.032–0.041)^27^. Although the results are difficult to compare as the PEARS trial was multi-component, our results do partly agree since we did not identify any associations between app usage (assessed as number of page views and app sessions) and diet quality or physical activity. However, we did identify statistically significant associations of the number of registrations with the change in diet quality and gestational weight gain. This suggests that different indicators of user engagement (app usage vs. registrations) could have different implications in the effectiveness of the app. Furthermore, registration of health behaviors (e.g., through goal setting and self-monitoring) has been suggested as an important behavior change technique in weight management^31^, also in pregnancy^32^. Furthermore, the lack of an association between user engagement and physical activity may also be viewed as intriguing. One reason for this finding could be that the HealthyMoms trial did not have any effect on physical activity which we previously hypothesized could partly be due to the late follow-up in pregnancy where the amount of physical activity generally has declined^9^. Another interesting finding was that it was mainly the physical activity registrations that were associated with lower gestational weight gain and greater diet quality. One reason for this finding could be that the physical activity registrations had greater variation and was the most used registrations feature with 62% of all registrations. In comparison, only 9% of the registrations were of diet. Indeed, our qualitative evaluation of the HealthyMoms app showed that some participants found the diet registration less motivating since it was difficult to remember dietary intakes and that feedback sometimes was ambiguous^30^. Furthermore, it is also relevant to consider differences in how registrations were conducted. For instance, the physical activity registrations, unlike diet and weight registrations, were only conducted when a “healthful” behavior had been performed which may explain why these registrations were underlying the associations with intervention effectiveness. It is also possible that the physical activity registration reflects a greater overall and deeper engagement with the app that other variables have not captured. Clearly, further studies are needed to elucidate the influence of different types of user engagement in exclusively digital interventions in pregnancy.

### Strengths and limitations

This study has several strengths such as the objective and accurate regarding user engagement (e.g., registrations, app usage and page views) and intervention outcomes (e.g., objectively and accurately measured weight gain and physical activity). Furthermore, the HealthyMoms trial had a very low drop-out rate (11% in the intervention group that was included in this study). Finally, another important strength is that we could adjust our results for a set of relevant confounders. For instance, it has been hypothesized that associations of user engagement in digital interventions with intervention effectiveness may be confounded by unmeasured factors^11^ such as baseline motivation, self-efficacy, or perceived competence which few previous studies have accounted for. For instance, women with higher motivation, self-efficacy or perceived competence may be less likely to have excessive gestational weight gain but may have greater interest to engage with an mHealth app. Thus, it is a strength of the study that we were able to adjust the estimates for baseline perceived competence of healthy diet and physical activity.

Our study also has several limitations that should be considered. First, our sample was generally highly educated and reported high baseline perceived competence for healthy diet and physical activity which may influence the generalizability of our findings. Although we adjusted our results for educational attainment and perceived competence, with little influence on the estimates, further studies are warranted in other populations. Furthermore, since the HealthyMoms intervention only reduced gestational weight gain in women with overweight or obesity before pregnancy, our ability to detect associations with gestational weight gain may have been limited although we were able to detect such associations. Finally, our sample size was relatively small (n = 55) regarding the extent of usage (i.e., app sessions and page views) which motivates further studies.

### Implications

Our results provide some evidence for beneficial associations of higher user engagement with the HealthyMoms app with lower gestational weight gain and improved diet quality. These results may be reconciled with previous results in non-pregnant populations which generally have shown associations between higher user engagement in digital interventions and weight loss^12^. Our results therefore indicate that high levels of user engagement are also desired in lifestyle interventions in pregnancy and that efforts could be made in order to increase user engagement in such interventions. However, although statistically significant, the identified associations in this study were generally of relatively small effect sizes which indicates that the results may be more relevant for larger groups of individuals rather than single individuals. Indeed, as previously hypothesized^11^ individuals may respond differently although engaging to a similar extent and the optimal dose of engagement may vary depending on user characteristics. More knowledge in this area may therefore enable the identification of individual engagement patterns that are beneficial for digital behavior change interventions. Thus, further studies are needed to examine whether different types of users respond differently to various levels and types of engagement in digital interventions in pregnancy.

## Conclusion

A greater number of total registrations (weight, diet and physical registrations) within the HealthyMoms app was associated with lower gestational weight gain and improved diet quality. However, the number of app sessions and page views had no associations with weight gain, diet quality and physical activity. Additional studies are needed to further elucidate the influence of different types of user engagement in digital pregnancy interventions on their effectiveness.

## Supplementary Information

Supplementary Information.

## Data Availability

The datasets used and analyzed during the current study are available from the corresponding author on reasonable request.
