# Supplementary material for: User engagement in relation to effectiveness of a digital lifestyle intervention (the HealthyMoms app) in pregnancy
Source: Sci Rep. 2022 Aug 13;12:13793. doi: 10.1038/s41598-022-17554-9 (PMC9376088; doi:10.1038/s41598-022-17554-9)
Supplement: Supplementary file 1 — Supplementary Information. [file 41598_2022_17554_MOESM1_ESM.pdf]

Supplementary material

User engagement in relation to effectiveness of a digital lifestyle intervention (the HealthyMoms app) in pregnancy

Pontus Henriksson* [1], Jairo H Migueles† [1,2], Emmie Söderström† [1,2], Johanna Sandborg [1,2], Ralph Maddison [3], Marie Löf [1,2,3]

[1] Department of Health, Medicine and Caring Sciences, Linköping University, Linköping, Sweden.

[2] Department of Biosciences and Nutrition, Karolinska Institutet, Huddinge, Stockholm, Sweden.

[3] Institute for Physical Activity and Nutrition, Deakin University, Geelong, Australia.

*** Corresponding author:** Pontus Henriksson, Department of Health, Medicine and Caring Sciences, Linköping University, 581 83 Linköping, Sweden. Telephone number: 013-28 10 00. E-mail: pontus.henriksson@liu.se

† These authors contributed equally

Table S1. Perceived Competence Scale for healthy diet and physical activity.

	Not at all true			Somewhat true			Very true
**Diet**							
1. I feel confident in my ability to make healthy food choices.	1	2	3	4	5	6	7
2. I am currently capable of making healthy food choices.	1	2	3	4	5	6	7
3. I am currently able to make my own healthy food choices.	1	2	3	4	5	6	7
4. I feel able to meet the challenge of making healthy food choices.	1	2	3	4	5	6	7
**Physical activity**							
1. I feel confident in my ability to be physically active regularly.	1	2	3	4	5	6	7
2. I am currently capable of being physically active regularly.	1	2	3	4	5	6	7
3. I am currently able to be physically active regularly.	1	2	3	4	5	6	7
4. I feel able to meet the challenge of being physically active regularly.	1	2	3	4	5	6	7

Table S2. Descriptive statistics for participants' characteristics in the intervention and the control group.

	Intervention[a]		Control[a]	
**Gestational week 14**	**n**	Value	**n**	Value
Age (y)	134	31.5 (4.2)	137	31.3 (3.9)
Educational attainment	134		137	
High school (12 y) or less, (% [n])		22.4 % (30)		19.0 % (26)
University degree, (% [n])		77.6 % (104)		81.0 % (111)
Parity	134		137	
0, (% [n])		58.2 % (78)		57.7 % (79)
≥ 1, (% [n])		41.8 % (56)		42.3 % (58)
Perceived competence for healthy diet (points)	134	21.9 (4.7)	137	22.2 (4.1)
Perceived competence for healthy PA (points)	134	20.3 (5.6)	137	21.0 (5.4)
Height (cm)	134	166 (6)	137	168 (6)
Weight (kg)	134	68.1 (12.9)	137	66.4 (9.5)
BMI (kg/m^2)	134	24.6 (4.3)	137	23.5 (3.0)
Swedish Healthy Eating Index score (points)[b]	133	6.51 (0.99)	135	6.81 (0.97)
Moderate-to-vigorous PA (min/day)[c]	130	38.8 (24.2)	134	40.3 (23.4)
**Gestational week 37**				
Weight (kg)	134	78.7 (13.1)	137	77.3 (10.6)
BMI (kg/m^2)	134	28.5 (4.2)	137	27.3 (3.4)
Swedish Healthy Eating Index score (points)	133	6.52 (0.93)	133	6.39 (1.07)
Moderate-to-vigorous PA (min/day)	131	26.3 (19.0)	135	27.8 (24.7)
**Change between gestational week 14 and 37**				
Gestational weight gain (kg)	134	10.6 (3.3)	137	10.8 (3.2)
BMI (kg/m^2)	134	3.8 (1.2)	137	3.8 (1.1)
Swedish Healthy Eating Index score (points)[b]	132	0.01 (1.10)	131	-0.42 (1.14)
Moderate-to-vigorous PA (min/day)[c]	127	-12.6 (20.9)	132	-12.8 (23.8)

BMI, body mass index; PA, physical activity; SD, standard deviation.

[a] Values are mean (SD) for continuous variables or % (n) for categorical variables.

[b] The Swedish Healthy Eating Index is based on 9 items 1) fruit and vegetables; 2) fish and shellfish; 3) red meat; 4) fiber; 5) wholegrain; 6) polyunsaturated fat; 7) monounsaturated fat; 8) saturated fat; 9) sucrose producing a score ranging from 0 to 9.

[c] Measured using accelerometry as described in the method section.

Table S3. User engagement over the 10 two-week themes.

		Theme									
**Engagement**	**Total**	**1**	**2**	**3**	**4**	**5**	**6**	**7**	**8**	**9**	**10**
**Number of app sessions**											
Median (IQR)	60 (46.5)	8 (6.5)	7 (6)	7 (8.5)	5 (8)	5 (5.5)	5 (5)	5 (6)	5 (5)	5 (5)	2 (5.5)
Any session (%)	100	100	100	98	96	93	91	91	89	82	62
**Number of page views**											
Median (IQR)	724 (771.5)	175 (193)	74 (122.5)	77 (83)	68 (79.5)	45 (60)	43 (66.5)	64 (86.5)	48 (69)	40 (48)	17 (57)
Any page view (%)	100	100	100	98	96	93	91	91	89	82	62
**Total registration**											
Median (IQR)	37.5 (94.75)	6 (9)	4 (10)	3 (9)	3 (10)	3 (8.75)	3 (8)	2 (9.75)	2 (8.75)	2 (7.75)	2 (6.75)
Any registration (%)	93	87	78	78	72	77	76	71	74	74	69
**Weight registrations**											
Median (IQR)	14 (16)	1 (1)	1 (2)	1 (2)	1 (2)	1 (2)	1 (2)	1 (2)	1 (2)	1 (2)	1 (2)
Any weight registration (%)	92	78	67	66	63	69	69	62	67	69	63
**Diet registrations**											
Median (IQR)	3 (9)	1 (2)	1 (2)	0 (1)	0 (1)	0 (1)	0 (1)	0 (0)	0 (0)	0 (0)	0 (0)
Any diet registration (%)	71	58	51	42	35	34	26	24	22	22	21
**PA registrations**											
Median (IQR)	13 (73.75)	2 (8)	1 (8)	0.5 (7)	0 (7)	0 (7)	0 (6)	0 (7)	0 (6)	0 (4.75)	0 (3.75)
Any PA registration (%)	70	62	56	50	49	49	44	42	37	36	32

IQR, interquartile range. PA, physical activity.

Table S4. Odds of excessive gestational weight gain per 1 SD increase in user engagement.

User engagement data	OR (95 % CI)	*P*
Total registrations		
Model 1[a]	0.74 (0.50, 1.08)	0.12
Model 2[b]	0.67 (0.44, 1.02)	0.060
Weight registrations		
Model 1[a]	1.04 (0.73, 1.48)	0.84
Model 2[b]	1.02 (0.71, 1.48)	0.92
Diet registrations		
Model 1[a]	0.89 (0.62, 1.28)	0.53
Model 2[b]	0.86 (0.59, 1.26)	0.45
Physical activity registrations		
Model 1[a]	0.68 (0.46, 1.02)	0.060
Model 2[b]	0.61 (0.39, 0.95)	0.029
Number of app sessions		
Model 1[a]	1.21 (0.66, 2.19)	0.54
Model 2[b]	1.10 (0.54, 2.23)	0.79
Number of page views		
Model 1[a]	0.95 (0.52, 1.73)	0.87
Model 2[b]	0.86 (0.43, 1.71)	0.67

[a] Adjusted for BMI at baseline.
[b] Adjusted for age, parity, educational attainment, baseline perceived competence for healthy diet and physical activity and baseline BMI.
